# Correspondence

**Published:** 1910-09

**Authors:** 

**Affiliations:** San Antonio, Texas


### Correspondence.

SAN ANTONIO, TEXAS, May 27, 1910.

*Dr. F. E. Daniel, Editor Texas Medical Journal, Austin, Texas.*

DEAR DOCTOR: I enjoy the "Red Back" very much, especially such articles as "Regulation of Marriage to Cut Down Ratio of Insanity," by Dr. Gregory. Whoop it up against tobacco and alcohol, the two main curses of the world. I can not agree, though, that sterilization is the only remedy. Tobacco should be wiped from the face of the earth by prohibitory laws, and alcohol taken over as the postage stamp, then sterilization would serve well its purpose. But a diagnosis without a remedy is of but little value; we must assist nature to abide by her—nature's—law. Inclosed find postoffice money order, hoping it will help to spread your business like good first-class Jersey butter on your pancakes.

Very respectfully,

DR. H. A. FEE AND WIFE.

